# Supplementary material for: National-scale spatiotemporal patterns of vegetation fire occurrences using MODIS satellite data
Source: PLoS One. 2024 Mar 28;19(3):e0297309. doi: 10.1371/journal.pone.0297309 (PMC10977707; doi:10.1371/journal.pone.0297309)
Supplement: Attachment — Submitted filename: Response to Editors request for edits.docx [file pone.0297309.s002.docx]

| **Comment** | **Response** |
| --- | --- |
| 1. We note that several of your files are duplicated on your submission. Please remove any unnecessary or old files from your revision, and make sure that only those relevant to the current version of the manuscript are included. | - Thank you for the comment. This has been corrected. Old and unnecessary files have been removed. |
| 2. Thank you for updating your data availability statement. You note that your data are available within the Supporting Information files, but no such files have been included with your submission. At this time we ask that you please upload your minimal data set as a Supporting Information file, or to a public repository such as Figshare or Dryad.  Please also ensure that when you upload your file you include separate captions for your supplementary files at the end of your manuscript.  As soon as you confirm the location of the data underlying your findings, we will be able to proceed with the review of your submission. | - Thank you for the comment. The authors have uploaded a minimal data set on Figshare. Please refer to the DOI: 10.6084/m9.figshare.24798327 - The location of the data has also been indicated in the manuscript (lines 457-458) - A zipped file of the data has also been uploaded to the Editorial manager. |
| 3. We note that Figures 1, 6 and 8 in your submission contain map images which may be copyrighted. All PLOS content is published under the Creative Commons Attribution License (CC BY 4.0), which means that the manuscript, images, and Supporting Information files will be freely available online, and any third party is permitted to access, download, copy, distribute, and use these materials in any way, even commercially, with proper attribution. For these reasons, we cannot publish previously copyrighted maps or satellite images created using proprietary data, such as Google software (Google Maps, Street View, and Earth). For more information, see our copyright guidelines: http://journals.plos.org/plosone/s/licenses-and-copyright.  We require you to either (1) present written permission from the copyright holder to publish these figures specifically under the CC BY 4.0 license, or (2) remove the figures from your submission: | - Thank you for your comment. - Please note that the maps presented in Figures 1, 6 and 8 are not copyrighted. The generation of the maps has been explained below. - Figures 1, 6 and 8 have been created by the authors using Zimbabwe's administrative boundaries. All the files utilized in the analysis are freely available. - Zimbabwe administration boundary (study area) was downloaded for free from <https://www.diva-gis.org/datadown> - Figure 1 refers to the study area showing the ecoregions extracted from the WWF global terrestrial ecoregions dataset which is freely available at <https://www.worldwildlife.org/publications/terrestrial-ecoregions-of-the-world> (*accessed on 15 March 2023*). The map in Figure 1 was created in ArcMap 10.5 using these freely available datasets (Zimbabwe administration boundaries and terrestrial ecoregions) - Figures 6 and 8 show maps that have been created through the emerging hotspot analysis method in ArcMap 10.5. The MODIS active fire data utilized in the emerging hotspot analysis was acquired freely from the Fire Information Management System (FIRMS) website <https://earthdata.nasa.gov/earth-observation-data/near-real-time/firms/active-fire-data> (accessed on 4 March 2023) as a shapefile (*shp). The fire data is provided by NASA for free. Refer to lines 114-133. - All the data sets used for analysis in this study are not copyrighted and have been provided freely. - Acknowledgement for the use of the data has been made in lines 454-458. |
| 4. Please ensure that you refer to Figure 7 in your text as, if accepted, production will need this reference to link the reader to the figure. | - Figure 7 has been referred to in lines 288-290 |
